# In step with strep

**DOI:** 10.1186/1757-1146-4-S1-P45

**Published:** 2011-05-20

**Authors:** Dimitra Psihramis, Luke Taylor, Nicholas Collins, Kenneth WK Ho

**Affiliations:** 1 High Risk Foot Clinic, Podiatry Department, Camden & Campbelltown Hospital, Sydney South West Area Health Service, Australia; 2 Macarthur Ambulatory Care Service, Camden & Campbelltown Hospital, Sydney South West Area Health Service, Australia; 3 High Risk Foot Clinic, Endocrinology Department, Camden & Campbelltown Hospital, Sydney South West Area Health Service, Australia

## 

A 47 year old man urgently presented to the outpatient High Risk Foot Clinic with an ulcerated right 5^th^ toe which had been present for two days and was deteriorating rapidly. On presentation, he had a wound measuring 40mm in diameter, at the base of the right fifth digit with cellulitis extending to the right knee. Swabs isolated growth of Group B Streptococcus and Staphylococcus Aureus organisms. X-rays were inconclusive for osteomyelitis. His medical history included Type 2 Diabetes Mellitus, hypertension, bilateral peripheral neuropathy and a history of neuropathic ulceration of the left foot. He was admitted to Campbelltown Hospital for IV antibiotic therapy, wound management and further investigations. On day two, the wound was debrided at the bedside and a bone scan was inconclusive for osteomyelitis. The cellulitus improved rapidly and retreated from the knee to the foot. On day four, a MRI showed inflammatory soft tissue changes without abscess formation, bone or joint involvement. He continued to improve clinically. A PICC line was inserted for ambulatory administration of IV cephazolin via Baxter infuser and he was discharged from hospital on day six. The patient was reviewed twice weekly by the podiatry, medical and nursing team from the High Risk Foot Clinic and Macarthur Ambulatory Care Service. During this time he had regular wound debridement and dressings, and 3 weeks of IV antibiotic therapy. He was then switched to 2 weeks of oral keflex. The patient returned to work 21 days from presentation and the wound completely healed 28 days from initial presentation.

